# Mechanical Chest Compression Device-Assisted Complex Percutaneous Coronary Intervention: A Life-Saving Approach for Persistent Ventricular Fibrillation During Coronary Intervention

**DOI:** 10.7759/cureus.97815

**Published:** 2025-11-25

**Authors:** Priyanka Deb Nath, Preethii Sasikala Palanivel, Wutyee Phoo, Girish Viswanathan

**Affiliations:** 1 Medicine, University Hospitals Plymouth NHS Trust, Plymouth, GBR; 2 Cardiology, University Hospitals Plymouth NHS Trust, Plymouth, GBR; 3 Acute Medicine, Derriford Hospital, Plymouth, GBR

**Keywords:** autopulse, cardiac death, effective resuscitation, mechanical chest compression device, pci, persistent ventricular arrhythmia

## Abstract

Sudden cardiac death remains a leading cause of mortality in acute coronary syndrome, driven largely by sustained ventricular arrhythmias (VAs) such as ventricular fibrillation (VF). Among patients with ST-elevation myocardial infarction (STEMI), 4-12% experience VAs within 48 hours of symptom onset. Persistent VF warrants prompt transport to a catheterization lab, and mechanical chest compression devices play a critical role in these cases. The AutoPulse, a battery-powered device with a load-distributing band, provides automated compressions that alleviate rescuer fatigue and allow uninterrupted medical focus on other vital tasks. By maintaining consistent perfusion throughout the percutaneous coronary intervention (PCI), the device facilitates life-saving interventions and highlights the potential utility of mechanical compression devices in rare, high-risk scenarios where conventional resuscitation techniques may be insufficient. This case report demonstrates the impact of the AutoPulse in achieving resuscitation and supporting primary percutaneous coronary intervention in a patient with resistant VF.

## Introduction

Sudden cardiac arrest is recognized as one of the leading causes of death, with patient survival to hospital discharge reported at only 2-9% [1]. The most frequent causes are myocardial infarction with refractory arrhythmia and pulmonary embolism [2]. Survival is largely dependent on adequately performed cardiopulmonary resuscitation (CPR) characterized by continuous, high-quality chest compressions and minimized interruptions, along with timely approached percutaneous coronary intervention (PCI) [2]. There are multiple challenges associated with performing prolonged manual CPR within the constraints of the catheterization laboratory, including limited space, radiation exposure risks, and rescuer fatigue, particularly during refractory arrests [3]. 

Mechanical chest compression devices deliver consistent compressions at the correct rate and depth, allow for complete chest recoil, and maintain high-quality CPR without being affected by rescuer fatigue [2]. They also permit safe defibrillation during resuscitation efforts [2]. Among the most commonly used devices are the AutoPulse (AutoPulse Resuscitation System Model 100, Zoll, CA) and the LUCAS (Lund University Cardiopulmonary Assist System, Jolife AB Inc., Lund, Sweden) [2-5]. Both devices have been shown to deliver high-quality chest compressions, although they operate based on different mechanical principles [2]. In the hands of a skilled and experienced operator, PCI can be safely continued, providing a potentially life-saving intervention during prolonged cardiac arrest [4].

## Case presentation

**Figure 1:**
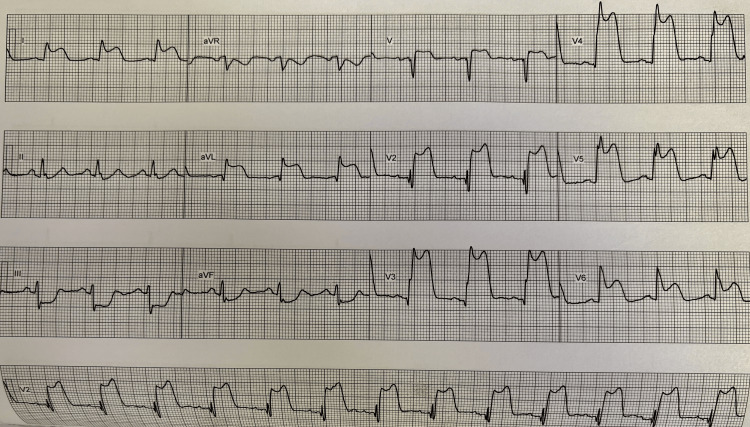
ECG showing ST elevation in V1-V6, I, and aVL.

A 66-year-old man who initially presented with severe chest pain became pale and diaphoretic at home. His past medical history includes long-standing hypertension and ex-smoker status. He has a family history of coronary artery disease, with his brother dying in his 50s and his mother in her 70s. The patient subsequently suffered an out-of-hospital ventricular fibrillation (VF) arrest but was reverted to sinus rhythm with a single shock by ambulance crew. He arrived at the hospital in cardiogenic shock with a blood pressure of 90/60 mmHg and an ECG demonstrating ST elevation in the anterolateral leads (Figure 1).

**Figure 2:**
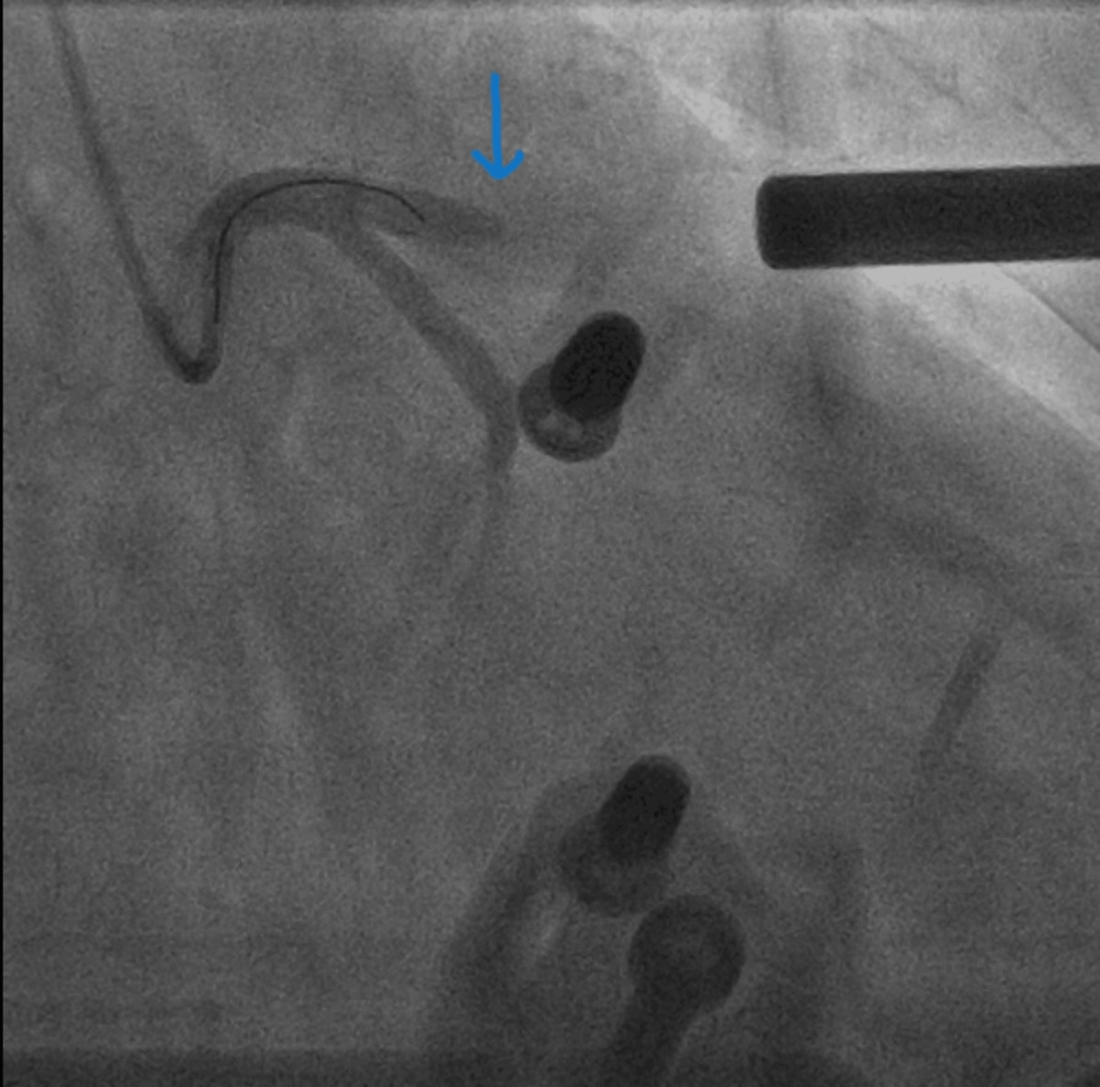
Culprit lesion blocked LAD. Blue arrow showing blocked LAD lesion. LAD: left anterior descending artery.

**Figure 3:**
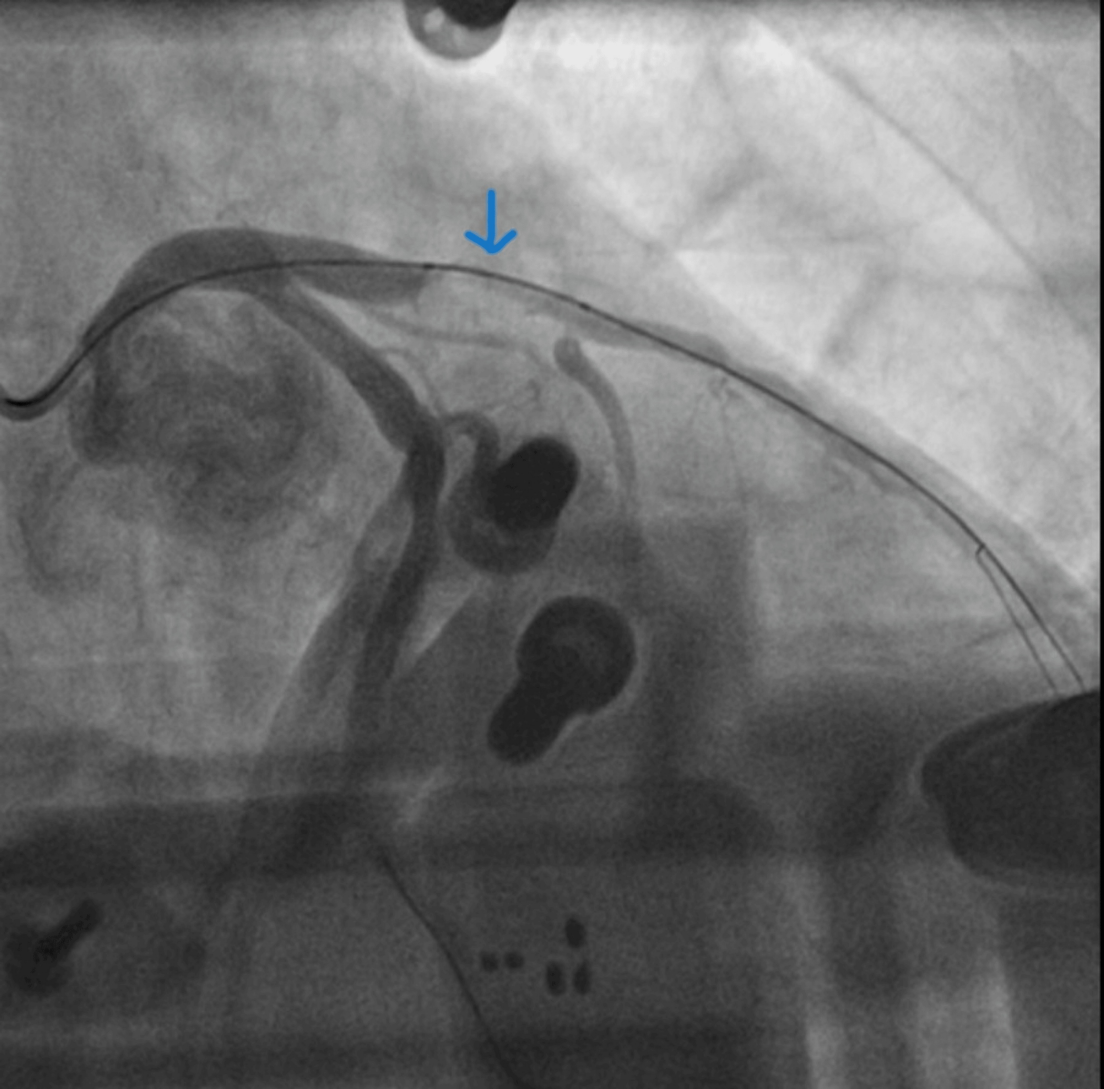
BMW wire was passed across the LAD. Blue arrow showing culprit LAD lesion after BMW wire passed through the LAD. LAD: left anterior descending artery.

**Figure 4:**
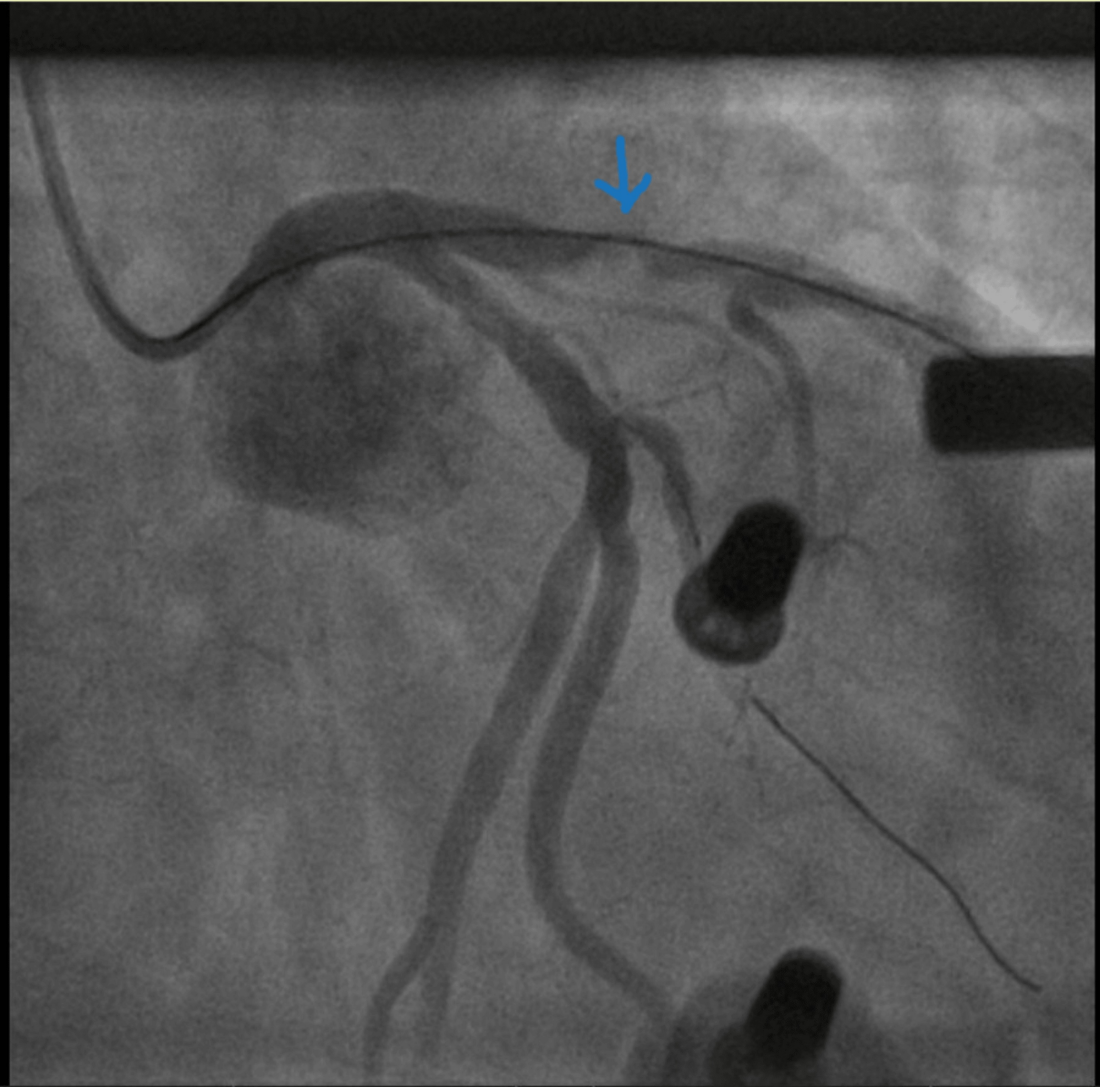
LAD flow occluded immediately. Blue arrow showing pre-dilated LAD using 2.0 × 15 mm, 2.5 × 15 mm and 1.5 × 15 mm Tazuna balloons, establishing TIMI 3 flow. However, the artery had immediate recoil, leading to immediate flow occlusion. LAD: left anterior descending artery.

**Figure 5:**
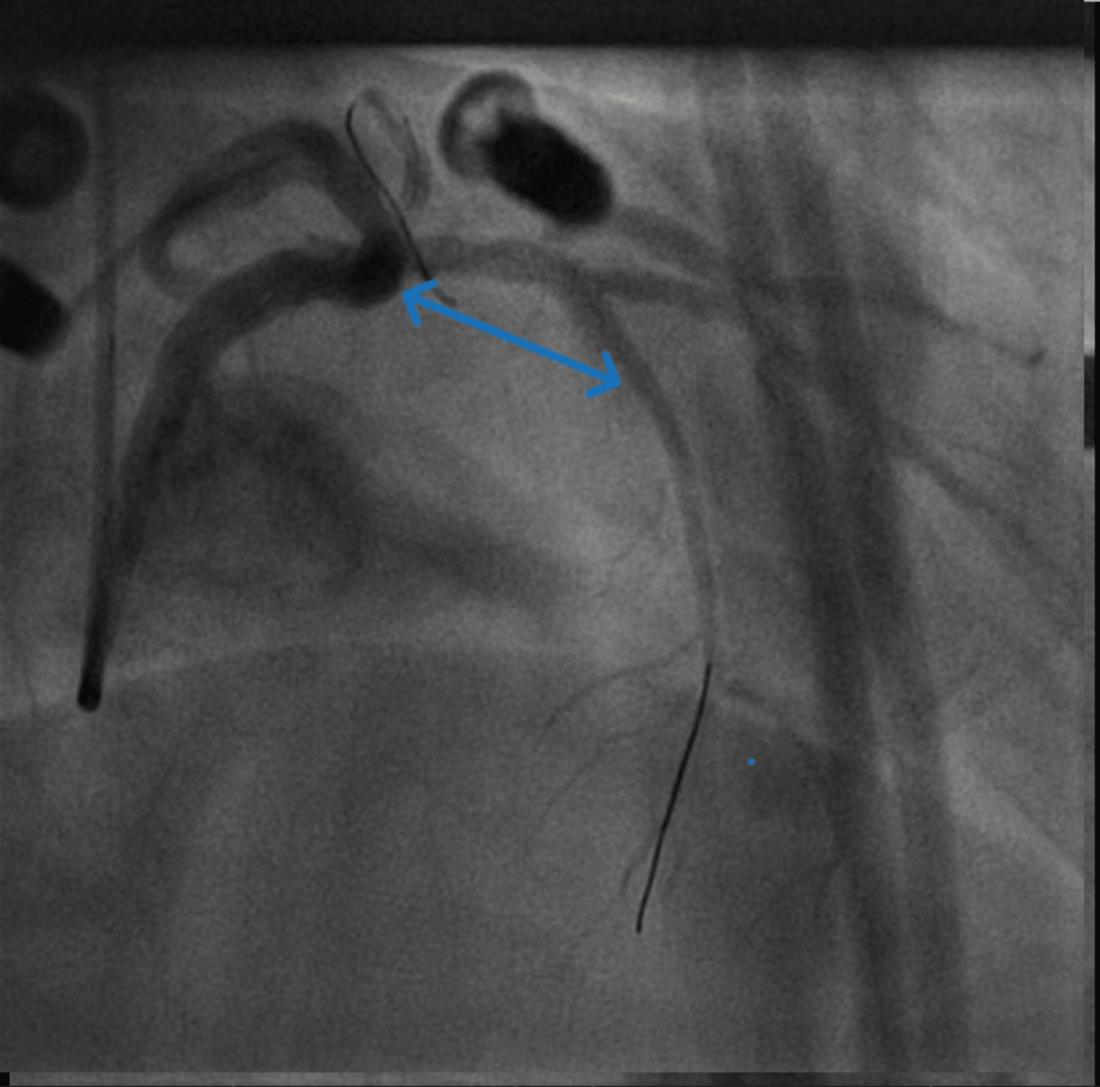
Drug-eluting stent was deployed and established TIMI 3 flow throughout the blocked LAD. Blue arrow showing the area of DES was deployed, establishing TIMI 3 flow. DES: drug-eluting stent; LAD: left anterior descending artery.

On admission, he was transferred to the cardiac catheterization laboratory. The patient had another episode of VF arrest. Initial treatment included 300 mg of Amiodarone followed by an additional 300 mg, and continued CPR was unsuccessful. Due to persistent VF arrest, under inotropic support, the AutoPulse device was deployed, providing continuous, effective mechanical chest compressions, which allowed the cardiac team to continue with the PCI. The initial angiographic findings showed a blocked left anterior descending artery (LAD) (Figure 2) and minimal atheroma in the left circumflex artery. A BMW wire was advanced across the circumflex to stabilize the guide catheter (Figure 3). Further, a BMW wire was passed across the LAD, which had heavy calcification and was proving difficult to cross the lesion. It was then swapped to a Fielder XT wire, which eventually crossed the lesion. He had a trifurcated LAD with a large septal branch (anatomical LAD), moderately large second diagonal, and a medium-sized first diagonal branch. The lesion was pre-dilated using 2.0 × 15 mm, 2.5 × 15 mm and 1.5 × 15 mm Tazuna balloons. This established Thrombolysis In Myocardial Infarction (TIMI) 3 flow. However, the artery had immediate recoil, leading to immediate flow occlusion (Figure 4). The lesion was further pre-dilated using a 2.5 × 15 mm non-compliant (NC) balloon. Further, a Sion wire was advanced into the anatomical LAD, and a 3.0 × 24 mm drug-eluting stent was successfully deployed, establishing TIMI 3 flow throughout the blocked LAD (Figure 5).

Post-PCI, the patient reverted to sinus rhythm and was transferred to the intensive care unit (ICU). Three days later, he was discharged on dual antiplatelet therapy consisting of aspirin and clopidogrel for 12 months, followed by aspirin to continue lifelong and other secondary prevention medications. The patient demonstrated progressive improvement, achieving full recovery at six-month follow up.

## Discussion

Persistent episodes of sustained ventricular tachycardia (VT), particularly in recurrent VF resistant to intervention may suggest incomplete reperfusion or the return of acute ischemia, warranting immediate coronary angiography. Although the primary goal in managing cardiac arrest is to restore coronary blood flow and reduce heart muscle damage, it is crucial to begin high-quality CPR immediately to ensure adequate perfusion of vital organs, especially the brain, until revascularization is possible [3]. 

Manual chest compressions have traditionally been used during cardiac arrest [4]. However, the introduction of mechanical chest compression devices presents several potential benefits, particularly in cases of prolonged resuscitation [4]. These devices can enhance fluoroscopic visibility during procedures, provide more consistent compressions reflected by improved aortic pressure waveforms, and offer ergonomic advantages for the medical team [4]. Additionally, they enable continuous and uninterrupted interventional procedures, which may be critical in life-saving situations [4]. The adoption of mechanical chest compression devices has been associated with a growing number of published case reports, reflecting increased clinical interest and utilization [4]. In the catheterization laboratory setting, these devices appear to improve the working conditions for staff and may provide more consistent and effective compressions compared to manual techniques [4].

Initial cardiac rhythm also plays a key role in survival after cardiac arrest [3]. Patients presenting with a shockable rhythm have significantly higher survival rates (32.6%) compared to those with non-shockable rhythms (11.4%), with nearly fivefold greater odds of survival (p < 0.001). This highlights the prognostic importance of the presenting rhythm as well [3].

The potential for injury associated with mechanical chest compression devices has been investigated in several studies. Smekal et al. compared 222 patients, with 83 receiving manual compressions and 139 treated with a mechanical device [6]. While rib and sternal fractures were common in both groups, the incidence of rib fractures was significantly higher in the mechanical group (78.8% vs. 64.6%, p = 0.01), whereas sternal fracture rates showed no significant difference (p = 0.555) [6]. Mediastinal and retrosternal hemorrhages were also more frequent in the mechanical group [6]. In a separate study, Ondruschka et al. reviewed autopsy findings from 614 patients after failed resuscitation attempts, comparing those who received manual chest compressions (501 patients, 81.6%) with those who received mechanical chest compressions (113 patients, 18.4%). No significant difference in overall injury severity was observed between manual and mechanical methods (p = 0.09) [7]. However, advanced age and longer resuscitation durations were linked to increased rates of thoracic and abdominal injuries, with mechanical compressions more often associated with hemothorax ( p= 0.047), pneumothorax (p = 0.008), hemopericardium (p = 0.025), and damage to the pulmonary (p = 0.008) and hepatic (p = 0.001) injuries [2-5,8].

In addition, social determinants of health also play a crucial role in cardiovascular outcomes. Access to mechanical chest compression devices is often limited in underserved or resource-constrained regions, which may adversely affect survival and recovery after cardiac arrest [9]. Socioeconomic challenges such as lower income, inadequate insurance coverage, and geographic barriers can further widen disparities in care [9]. Recognizing and addressing these inequities are essential to improve access to life-saving interventions and reduce outcome disparities in high-risk cardiac patients.

This case highlights the role of the AutoPulse in providing high-quality, uninterrupted chest compressions during prolonged resuscitation and complex procedures. The device’s ability to maintain consistent perfusion during PCI has the potential to contribute significantly to the patient survival.

## Conclusions

Mechanical chest compression devices may offer important advantages over manual compressions during prolonged cardiac arrest in the cardiac catheterization lab. Their use allows critical interventions to continue during CPR, which may help improve patient outcomes. This case is particularly unique because it demonstrates the successful use of a mechanical chest compression device to maintain continuous CPR during a complex PCI procedure in a patient with persistent ventricular fibrillation. This scenario is both rare and challenging to manage. It highlights how such devices can facilitate life-saving interventions in situations where manual compressions alone may be insufficient, offering valuable insights for similar high-risk clinical scenarios. Given their reasonable cost, devices like the AutoPulse could be made available in more emergency settings, including ambulances. However, more research is needed to clearly define their role in frontline emergency care.
