# Correction: Mechanical Chest Compression Device-Assisted Complex Percutaneous Coronary Intervention: A Life-Saving Approach for Persistent Ventricular Fibrillation During Coronary Intervention

**DOI:** 10.7759/cureus.c424

**Published:** 2026-04-15

**Authors:** Priyanka Deb Nath, Preethii Sasikala Palanivel, Wutyee Phoo, Girish Viswanathan

**Affiliations:** 1 Medicine, University Hospitals Plymouth NHS Trust, Plymouth, GBR; 2 Cardiology, University Hospitals Plymouth NHS Trust, Plymouth, GBR; 3 Acute Medicine, Derriford Hospital, Plymouth, GBR

This article has been corrected to add Girish Viswanathan as last author. The authors deeply regret the oversight.

